# Supplementary material for: How pharmacy and medicine students experience the power differential between professions: “Even if the pharmacist knows better, the doctor’s decision goes”
Source: PLoS One. 2021 Aug 26;16(8):e0256776. doi: 10.1371/journal.pone.0256776 (PMC8389418; doi:10.1371/journal.pone.0256776)
Supplement: Attachment — Submitted filename: Response to Reviewers.docx [file pone.0256776.s001.docx]

Dear PLOS One Editor and reviewers,

Re: How pharmacy and medicine students experience the power differential between professions: “even if the pharmacist knows better, the doctor’s decision goes”

Thank-you for asking us to address the following:

| 1. Please ensure that your manuscript meets PLOS ONE's style requirements, including those for file naming. | Formatting has been revised to comply with journal requirements. Title page added. References have been checked. |
| --- | --- |
| 2. Please include additional information regarding the interview guide used in the study and ensure that you have provided sufficient details that others could replicate the analyses. For instance, if you developed an interview guide as part of this study and it is not under a copyright more restrictive than CC-BY, please include a copy, in both the original language and English, as Supporting Information. | The interview prompt questions have now been included in the supporting information. |
| 3. Please provide additional details regarding participant consent. In the ethics statement in the Methods and online submission information, please ensure that you have specified whether consent was informed. | Participants were provided with an information sheet and completed a written consent form (lines 132-133). This information has now been included in the manuscript, specifically the statement that “students gave informed consent for interview” (line 116).  Participant information sheet and consent form also provided in supporting information |
| 4. We note that you have indicated that data from this study are available upon request. PLOS only allows data to be available upon request if there are legal or ethical restrictions on sharing data publicly. For more information on unacceptable data access restrictions, please see http://journals.plos.org/plosone/s/data-availability#loc-unacceptable-data-access-restrictions  In your revised cover letter, please address the following prompts:  a) If there are ethical or legal restrictions on sharing a de-identified data set, please explain them in detail (e.g., data contain potentially sensitive information, data are owned by a third-party organization, etc.) and who has imposed them (e.g., an ethics committee). Please also provide contact information for a data access committee, ethics committee, or other institutional body to which data requests may be sent. | The research participants for this study did not provide consent for data sharing. Unfortunately, it is not possible to retrospectively obtain consent to allow us to share the data publicly. The University of Adelaide, School of Psychology, Human Research Ethics Subcommittee AND the Flinders University Social and Behavioural Research Ethics Committee have approved the research protocol with the specified conditions:  -The data will only be accessible to the researchers.  -It is anticipated that the results from this study will be published in a peer reviewed article. No participants will be identified in the publication and only aggregated data will be published.  Contact for The University of Adelaide, School of Psychology, Human Research Ethics Subcommittee: ph 61 8 8313 5693, fax 61 8 8313 3770, email: [psychologyoffice@adelaide.edu.au](mailto:psychologyoffice@adelaide.edu.au)  Full names of ethics committees provided in manuscript (at lines 129-131) |
| 5. Reviewer #1: well planned and executed study. how do you made sure that all the participants attended IPE workshops? What's your recommendations to improve the collaboration between medicine and pharmacy professionals? | Non-attendance was an infrequent event. However, both the recruitment notice and the information sheet clearly outlined that attendance at workshops was a prerequisite for participation in the research activity. Additionally, the interview included an opening statement and question about the workshops. Provision of the interview questions and participant information sheet in the supporting information, clarifies this.  Our recommendations are for educators to facilitate discussion about professional identity, power, hierarchy in health professions curricula. Additional sentence added to conclusion. We have also advocated for greater institutional support through policy and accreditation, and further clarified this statement in the discussion (lines 381- 383 and 392-393). |
| Reviewer #2: Review Comments to the Author The sample size included in the study was very small. How would the author clarify this point? | Despite the small sample size, the data obtained from the interviews are rich and deep, offering a strong narrative around the research question. In addition, there was an element of data triangulation achieved from our previous research (which utilised students’ written reflections), as well as triangulation of perspectives across multiple researchers. This adds to the credibility of the findings in line with the quality criteria for qualitative research.  We have included a paragraph at the end of the discussion section (lines 399-403)  Ref. Frambach, J. M., van der Vleuten, C. P., & Durning, S. J. AM last page: Quality criteria in qualitative and quantitative research. Academic Medicine. 2013,88(4), 552. |

Yours sincerely,

A/Prof. Josephine Thomas,

Corresponding Author

[Josephine.thomas@adelaide.edu.au](mailto:Josephine.thomas@adelaide.edu.au)
